# Potential predictors of susceptibility to occupational stress in Japanese novice nurses - a pilot study

**DOI:** 10.1186/s12199-017-0641-8

**Published:** 2017-04-04

**Authors:** Shinobu Okita, Satoshi Daitoku, Masaharu Abe, Emi Arimura, Hitoshi Setoyama, Chihaya Koriyama, Miharu Ushikai, Hiroaki Kawaguchi, Masahisa Horiuchi

**Affiliations:** 1 Department of Hygiene and Health Promotion Medicine, Kagoshima University Graduate School of Medical and Dental Sciences, Kagoshima, 890-8544 Japan; 2 Department of Life and Environmental Science, Kagoshima Prefectural College, Kagoshima, Japan; 3 Center for Education of Medical Residents, Kagoshima University Hospital, Kagoshima, Japan; 4 Department of Epidemiology and Preventive Medicine, Kagoshima University Graduate School of Medical and Dental Sciences, Kagoshima, Japan

**Keywords:** Body weight change, Brief job stress questionnaire, Employee turnover, Pre-employment health examinations, Urinary minerals

## Abstract

**Background:**

Occupational stress is a known factor behind employee resignations; thus, early identification of individuals prone to such stress is important. Accordingly, in this pilot study we evaluated potential predictors of susceptibility to occupational stress in Japanese novice nurses.

**Methods:**

Forty-two female novice nurses at Kagoshima University Hospital were recruited for the study population. Each underwent physical health and urinary examinations, and completed a lifestyle questionnaire at the time of job entry. Each also completed a Brief Job Stress Questionnaire (BJSQ), related to mental health status, at job entry and 5 months post-entry. Psychological stress, somatic symptoms, and combined BJSQ scores were determined for each time point.

**Results:**

All three stress condition scores had significantly decreased at 5 months post-entry, suggesting occupational stress. Systolic blood pressure (*r* = −0.324, *p* < 0.05) and urinary sodium (*r* = −0.313, *p* < 0.05) were significantly negatively correlated with combined BJSQ score at 5 months post-entry. Post-entry stress condition scores were significantly low in subjects reporting substantial 1-year body weight change (≤ ± 3 kg) and short times between dinner and bedtimes (≤2 h), though baseline stress condition scores were not. Urinary sodium concentration, 1-year body weight change, and pre-sleep evening meals were then targeted for multivariate analysis, and confirmed as independent explanatory variables for post-entry stress condition scores.

**Conclusions:**

One-year body weight change, times between dinner and bedtimes, and urinary sodium concentration are promising potential predictors of susceptibility to occupational stress, and should be further investigated in future research.

**Trial registration:**

ISRCTN ISRCTN17516023. Retrospectively registered 7 December 2016.

## Background

Occupational stress is known as an important factor affecting workers’ physical and mental health [1–3]. Overly stressful conditions may result in resignations and subsequently increased employee turnover [4, 5]. Research in Japan’s nursing sector has found that protecting new employees from occupational stress is important for reducing such turnover [6, 7]. When new employees with potentially high susceptibility to occupational stress are identified at an early stage, it is easier to safeguard them from stressors and potentially stressful situations. Intrinsic, pre-employment factors are known to play an important role in stress in addition to extrinsic, work-related factors [8–10], suggesting occupational stress forms a distinct part of an individual’s stress burden. Accordingly, methods to evaluate individual susceptibility to occupational stress are clearly beneficial for formulating strategies to reduce employee turnover. However, such methods remain to be elucidated.

A self-administered Brief Job Stress Questionnaire (BJSQ) forms the basis of mental assessments in statutory annual health examinations for Japanese workers. These assessments focus on occupational stress, and since 2015 have been legally required in Japan in addition to physical health assessments. The BJSQ was originally developed by the Ministry of Health, Labour and Welfare, Japan and contains 57 items classified into three categories: A (measurement for job stressors), B (measurement for psychophysical complaints), and C (measurement for support for workers) [11]. Questions in category B are used to evaluate psychological and physical health conditions related to overall mental health. The BJSQ is widely used to evaluate employees’ mental health status, and category B has been used to investigate workplace stress and its impact on mental health [2, 11, 12]. Such stress analyses are based on demand-control-support and effort-reward-imbalance models [13].

Data accumulated from the BJSQ facilitate identification of individuals with high occupational stress. This stress can then be ameliorated by managing stressors identified through the questionnaire models, as well with heightened social support from supervisors and colleagues [14]. Specific predictions of susceptibility to occupational stress based on the data enable supervisors and managers to make more effective interventions. Any such predictive analysis needs to take account of differences in intrinsic, pre-employment factors, because occupational stress levels are known to differ among individuals with similar workplace conditions and workloads [8–10].

In the present study, we aimed to identify potential predictors of susceptibility to occupational stress. We investigated items related to lifestyles and eating habits, along with physical health examination parameters. As urinary parameters, sodium, potassium, and phosphorus excretions were also included in our evaluation, as they may be related to oral mineral intake [15]. The stress conditions of each study subject were determined at job entry and 5 months post-entry in the form of scores derived from the subjects’ BJSQ responses. Obtained data were then analysed to determine the presence of any relationships between the stress condition scores and the above-stated potential predictors (lifestyle items, eating habits, and physical health and urinary examination parameters), both at job entry and 5 months post-entry.

## Methods

### Subjects

**Fig. 1:**
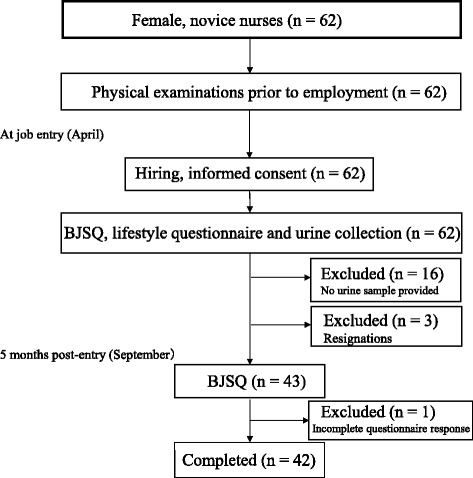
Study subjects selection and exclusion. The *bold-bordered square* indicates the entire initial population enrolled in the study

The subjects in the present study were recruited from among 86 nurses who began employment at Kagoshima University Hospital, Japan, in April 2015. Sixty-two satisfied the inclusion criteria (female, novice-level nurses). The study was approved by the institutional review committee of Kagoshima University Hospital, and all participants gave informed consent (No. 26–161). Before employment, all 62 nurses had undergone the mandatory health examinations required under the Japanese Industrial Safety and Health Act, and were asked to complete category B of the BJSQ to evaluate their stress levels at job entry. They were also asked to provide urine samples 2 weeks thereafter. Sixteen did not provide samples and, thus, were excluded from the study. At 5 months post-entry (September 2015), subjects were again asked to complete category B questions of the BJSQ. Four subjects were excluded from the study in September: three because of resignation and one for an incorrectly completed questionnaire (Fig. 1). Accordingly, the final analysis population consisted of 42 subjects. During the first 5 months of their employment at Kagoshima University Hospital, the nurses in this study were primarily involved in training on patient care procedures; therefore, job conditions and content did not differ greatly among study subjects.

### Physical health examination, education level, and area of residence

Each subject underwent an individual physical health examination before job entry. Body weight, height, and systolic and diastolic blood pressures were measured as standard parameters (Table 1). Body mass index was calculated with the standard equation. Red blood cell count, hemoglobin concentration, aspartate transaminase (AST), alanine transaminase (ALT), γ-glutamyltranspeptidase (γ-GTP), low-density lipoprotein (LDL) cholesterol, high-density lipoprotein (HDL) cholesterol, and triglycerides were measured as biochemical parameters. Additionally, each nurse’s level of educational attainment (university or vocational college) and area of residence for higher education (within or outside Kagoshima Prefecture, in which the hospital is located) were recorded.

**Table 1:** Population characteristics at job entry (start of study)

		N	Mean		SD	Minimum	Maximum
Education and area of residence							
Level of higher education	University, vocational college	37/5					
Area of residence for higher education	Kagoshima Prefecture, outside Kagoshima Prefecture	35/7					
Physical health examination							
Age, years			21.9	±	0.6	21	24
Body mass index, kg/m^2^			20.2	±	2.4	17.1	26.8
Systolic BP, mm Hg			111.5	±	11.6	93	144
Diastolic BP, mm Hg			67.6	±	9.5	46	88
Hemoglobin, g/dL			13.3	±	0.9	10.8	15.4
AST, U/L			18.1	±	5.1	11	41
ALT, U/L			15.4	±	10.3	6	66
γ-GTP, U/L			16.3	±	7.6	7	46
LDL cholesterol, mg/dL			96.7	±	21.9	56	150
HDL cholesterol, mg/dL			69.3	±	11.7	41	91
Triglycerides, mg/dL			58.7	±	22.1	25	113
Fasting blood glucose, mg/dL			86.9	±	6.5	68	99
Urinary parameters							
Sodium, mg/mg Cr			2.59	±	1.58	0.71	8.86
Potassium, mg/mg Cr			0.83	±	0.46	0.31	2.42
Chlorine, mg/mg Cr			2.05	±	1.97	0.35	12.16
Phosphorus, mg/mg Cr			0.64	±	0.14	0.29	0.94
Calcium, mg/mg Cr			0.08	±	0.05	0.01	0.22
Magnesium, mg/mg Cr			0.06	±	0.03	0.01	0.12
Questionnaire on Lifestyle							
Smoking	Non-smoker/Current smoker	42/0					
Alcohol consumption	Never/Once a week/Every day	12/30/0					
Body weight change since age of 20; ≥ 10 kg	No/Yes	42/0					
One-year body weight change; ≥ 3 kg	No/Yes	21/21					
Exercise							
Exercise habit	No/Yes	42/0					
Walking habit	No/Yes	30/12					
Fast walking habit	No/Yes	22/20					
Eating							
Fast eating speed	No/Yes	26/16					
Short time between dinner and bedtimes (<2 h)	No/Yes	10/32					
Snack after dinner	No/Yes	23/19					
Breakfast skipping	No/Yes	28/14					

Values are expressed as the mean ± SD or number (N) of the subjects. *BP* blood pressure, *AST* aspartate transaminase, *ALT* alanine transaminase, *γ-GTP* γ-glutamyltranspeptidase, *LDL* low-density lipoprotein, *HDL* high-density lipoprotein, *Cr* creatinine

### BJSQ and lifestyle questionnaire

Individual mental health status was evaluated using responses to BJSQ category B, with a method outlined in previously published research [2, 11, 16]. Category B concerns psychological and physiological aspects of mental health status, and responses to questions in that category were used to determine a psychological stress condition score, somatic symptom condition score, and combined BJSQ score (psychological stress + somatic symptom condition scores). Lower condition scores represented a higher stress level. Study subjects completed the same self-administered questionnaire at job entry (April) and again at 5 months post-entry (September).

The subjects were also asked to complete a lifestyle questionnaire similar to those commonly used in medical examinations [17], at job entry. The questionnaire included questions on current habits and pre-employment lifestyle and body weight. It contained 11 items: anti-hypertensive drug, anti-diabetic drug, and/or anti-dyslipidemia drug medication history; stroke, coronary heart disease, kidney disease, and/or anemia history; smoking habit; alcohol habit; body weight change (≤ ±10 kg) since age 20; body weight change (≤ ±3 kg) since previous annual health examination; exercise habit; walking speed; eating speed; times between dinner and bedtimes (short: ≤ 2 h; ≥ 2 days/week), eating snacks after evening meals (≥2 days/week); and skipping breakfast (≥2 days/week).

### Urinary examinations

Each study subject was asked to provide a morning urine sample within the first 2 weeks of employment. Urinalysis was commissioned to a laboratory (Clinical Pathology Laboratory, Co. Ltd., Kagoshima, Japan), where the samples were analysed for sodium, potassium, chlorine, phosphorus, calcium, and magnesium by standard methods. The obtained concentrations were divided by the creatinine content to yield values reported to predict total amounts excreted in urine in 24 h [18].

### Data analysis

Change in BJSQ score between job entry (April) and 5 months post-entry (September) was analysed using the Wilcoxon signed-rank test. Physical health examination and urinary parameter results are shown as mean ± standard deviation (SD). Simple correlations between BJSQ score, and each physical health examination parameter, urinary parameter, or nominal variable of the lifestyle questionnaire were evaluated with the Spearman’s rank correlation coefficient. Stress condition scores at job entry and 5 months post-entry were analysed against nominal variables of lifestyle questionnaire with the Mann–Whitney *U* test or Kruskal–Wallis test, as appropriate. Urinary sodium, 1-year (pre-employment) body weight change (≤ ±3 kg), and times between dinner and bedtimes (≤2 h) were selected as potential explanatory variables for stress condition scores (objective variables) at 5 months post-entry in multiple regression analysis after checking for multicollinearity. The level of significance was set at *p* < 0.05. Stata 14 (StataCorp LP, College Station, TX) software was used for statistical analysis when appropriate.

## Results

### Study population characteristics

The study population was selected as shown in Fig. 1, and comprised 42 female novice nurses. A total of 62 nurses were initially enrolled, gave informed consent, and completed physical health examinations, lifestyle questionnaires, and the BJSQ at job entry, and the final study population of 42 nurses represented subjects not excluded in the analysis period. Of these 42, 37 graduated from university and five from vocational colleges. Subject ages ranged 21–24 (mean, 21.9) years (Table 1).

### Physical health and urinary examination and lifestyle questionnaire results at job entry

Table 1 shows the physical health examination and lifestyle questionnaire results. No subjects were current smokers. No subjects reported body weight change since age 20 that was > ± 10 kg or ± 10 kg. However, 21 subjects reported 1-year (pre-employment) body weight change > ± 3 kg or ± 3 kg (change since previous annual health examination). Thirty-two subjects reported they had short times between dinner and bedtimes (≤2 h) on ≥ 2 days/week. Fourteen subjects reported skipping breakfast on ≥ 2 days/week. Table 1 shows creatinine-corrected results for mineral concentrations in spot urine samples.

### Stress condition scores at job entry and 5 months post-entry

**Fig. 2:**
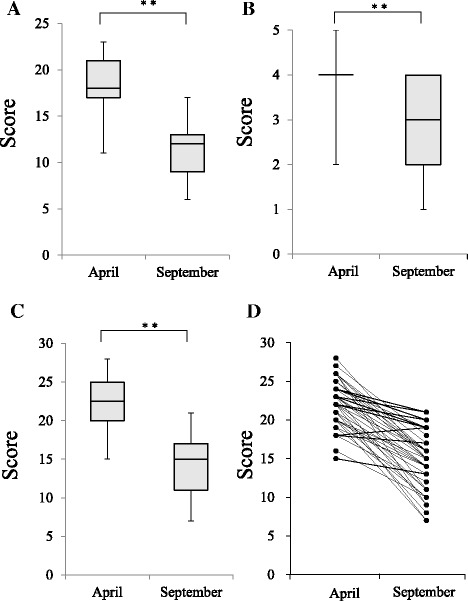
Psychological stress and somatic symptom condition score and combined Brief Job Stress Questionnaire (BJSQ) score between baseline and 5 months post-entry. Psychological stress condition score (**a**), somatic symptom condition score (**b**) and combined BJSQ score (**c**), and individual combined BJSQ scores (**d**) are shown for job entry (April, baseline) and 5 months post-entry (September). The data (**a**–**c**) are shown as maximum, 75%, median, 25%, and minimum as a boxplot. **p < 0.01 values was regarded as statistically significant in the Wilcoxon signed-rank test

Stress condition scores (derived from BJSQ responses) obtained at both time points are shown as box plots (Fig. 2); lower scores represent higher stress levels. Psychological stress and both somatic symptom condition scores and combined BJSQ score were significantly decreased at 5 months after starting employment (Fig. 2, box plots A–C). Individually, 41 of the 42 subjects showed a decreased combined BJSQ score at 5 months post-entry (Fig. 2, box plot D).

### Correlations between stress condition scores and possible stress predictors in univariate analyses of BJSQ responses

- At baseline
  Baseline stress conditions showed no significant correlation with any physical health or urinary examination parameter or nominal variable of the lifestyle questionnaire (Tables 2 and 3, upper sections), with the exception of eating speed. Psychological stress condition and combined BJSQ scores were significantly lower in subjects who reported a fast eating speed.

**Table 2:** Correlation between stress conditions scores at job entry (A) and 5 months post-entry (B) with physical examination and urinary parameters at job entry

Continuous variables	Psychological stress condition score		Somatic symptom condition score		Combined BJSQ score	
	Correlation	*p* value	Correlation	*p* value	Correlation	*p* value
A. April (at job entry)						
Physical examination (at job entry)						
Body mass index	−0.161	0.308	−0.090	0.570	−0.168	0.289
Systolic BP	0.071	0.657	0.127	0.424	0.082	0.606
Diastolic BP	−0.133	0.400	0.024	0.879	−0.120	0.449
Hemoglobin	0.000	1.000	−0.170	0.283	−0.035	0.825
AST	0.050	0.753	−0.109	0.493	0.003	0.985
ALT	−0.011	0.946	−0.065	0.685	−0.048	0.764
γ-GTP	−0.255	0.103	0.031	0.844	−0.218	0.166
LDL cholesterol	0.154	0.332	0.071	0.654	0.168	0.287
HDL cholesterol	0.045	0.778	0.176	0.266	0.072	0.650
Triglycerides	−0.177	0.262	−0.131	0.407	−0.176	0.266
Fasting blood glucose	−0.148	0.349	−0.216	0.170	−0.194	0.218
Urinary parameters						
Sodium	0.135	0.393	−0.164	0.299	0.057	0.721
Potassium	0.247	0.115	−0.109	0.492	0.168	0.288
Chlorine	0.171	0.279	−0.137	0.385	0.096	0.546
Phosphorus	−0.109	0.490	−0.199	0.208	−0.155	0.327
Calcium	0.192	0.224	0.186	0.239	0.198	0.208
Magnesium	0.076	0.633	0.016	0.920	0.078	0.624
B. September (5 months post − entry)						
Physical examination (at job entry)						
Body mass index	−0.125	0.432	−0.126	0.427	−0.126	0.428
Systolic BP	−0.335	0.030*	−0.210	0.183	−0.324	0.036*
Diastolic BP	−0.136	0.390	−0.109	0.493	−0.147	0.352
Hemoglobin	−0.129	0.415	−0.251	0.109	−0.166	0.293
AST	0.027	0.866	−0.091	0.565	−0.005	0.973
ALT	0.066	0.680	0.004	0.978	0.047	0.770
γ-GTP	−0.015	0.925	−0.164	0.299	−0.077	0.630
LDL cholesterol	0.159	0.316	−0.080	0.615	0.102	0.520
HDL cholesterol	0.212	0.178	0.102	0.520	0.168	0.288
Triglycerides	0.138	0.383	0.078	0.623	0.148	0.349
Fasting blood glucose	0.051	0.747	−0.072	0.650	0.006	0.971
Urinary parameters						
Sodium	−0.284	0.068^#^	−0.264	0.091^#^	−0.313	0.043*
Potassium	−0.107	0.499	0.030	0.851	−0.090	0.570
Chlorine	−0.202	0.200	−0.116	0.465	−0.199	0.206
Phosphorus	−0.286	0.066^#^	−0.249	0.112	−0.301	0.053^#^
Calcium	−0.161	0.308	0.059	0.710	−0.114	0.473
Magnesium	−0.082	0.605	0.048	0.765	−0.054	0.734

Values are expressed as the mean ± SD or number (N) of the subjects. *BP* blood pressure, *AST* aspartate transaminase, *ALT* alanine transaminase, *γ − GTP* γ-glutamyltranspeptidase, *LDL* low-density lipoprotein, *HDL* high-density lipoprotein. ^#^ *p* < 0.10; *p < 0.05

**Table 3:** Comparison of stress conditions scores at job entry (A) and 5 months post-entry (B) with nominal variables of lifestyle questionnaire at job entry

Nominal variables		*N*	Psychological stress condition score		Somatic symptoms condition score		Combined BJSQ score	
			Median (25%, 75%)	*p* value	Median (25%, 75%)	*p* value	Median (25%, 75%)	*p* value
A. April (at job entry)								
Education level	Vocational college	5	17 (17, 18)	0.137	4 (3, 4)	0.581	21 (20, 21)	0.127
	University	37	19 (17, 21)		4 (4, 4)		23 (20, 25)	
Area of residence for higher education	Kagoshima Prefecture	35	18 (15, 18)	0.059	4 (4, 4)	0.555	22 (19, 23)	0.110
	outside Kagoshima Prefecture	7	19 (17,21)		4 (3, 5)		23 (20, 26)	
Alcohol consumption	Never	12	18 (17.5, 20.5)	0.900	4 (3, 4)	0.274	22 (20.5, 24.5)	0.801
	Once a week	30	19 (16, 21)		4 (4, 5)		23 (20, 25)	
	Every day	0	0 (0, 0)		0 (0, 0)		0 (0, 0)	
One-year body weight change > 3 kg	No	21	18 (17, 20)	0.810	4 (4, 5)	0.442	23 (20, 24)	0.879
	Yes	21	18 (17, 21)		4 (3, 4)		22 (20, 25)	
Exercise								
Walking habit	No	30	18.5 (17, 21)	0.674	4 (4, 4)	0.867	23 (21, 24)	0.537
	Yes	12	18 (16, 21)		4 (3.5, 4.5)		21 (19.5, 25.5)	
Fast walking habit	No	22	19 (16, 21)	0.929	4 (3, 4)	0.441	23 (20, 25)	0.939
	Yes	20	18 (17.5, 20.5)		4 (4, 5)		22 (20.5, 24)	
Eating								
Fast eating speed	No	26	19.5 (18, 21)	0.011*	4 (4, 4)	0.282	23.5 (22, 26)	0.010*
	Yes	16	17.5 (16, 18.5)		4 (3, 4.5)		21 (19.5, 22.5)	
Short time between dinner and bedtimes (<2 h)	No	10	18.5 (16, 21)	0.812	4 (3, 5)	0.987	23 (19, 24)	0.894
	Yes	32	18 (17, 21)		4 (4, 4)		22 (20.5, 25)	
Snack after dinner	No	23	19 (18, 21)	0.147	4 (4, 4)	0.385	23 (20, 26)	0.186
	Yes	19	18 (16, 20)		4 (3, 5)		22 (20, 23)	
Breakfast skipping	No	28	19 (17, 21)	0.188	4 (4, 4.5)	0.662	23 (20.5, 25.5)	0.217
	Yes	14	18 (16, 20)		4 (3, 4)		21.5 (20, 24)	
B. September (5 months post-entry)								
Education level	Vocational college	5	11 (10, 12)	0.653	3 (2, 3)	0.936	14 (13, 14)	0.572
	University	37	12 (9, 13)		3 (2, 4)		15 (11, 17)	
Area of residence for higher education	Kagoshima Prefecture	35	12 (9, 13)	0.987	3 (2, 4)	0.598	15 (11, 17)	0.933
	outside Kagoshima Prefecture	7	11 (9,15)		3 (2, 4)		14 (11, 19)	
Alcohol consumption	Never	12	11.5 (7.5, 14.5)	0.614	2.5 (2, 3.5)	0.417	14.5 (9, 17.5)	0.665
	Once a week	30	12 (9, 13)		3 (2, 4)		15 (12, 17)	
	Every day	0	0 (0, 0)		0 (0, 0)		0 (0, 0)	
One-year body weight change > 3 kg	No	21	13 (11, 15)	<0.01**	3 (3, 4)	0.015*	16 (15, 19)	<0.01**
	Yes	21	9 (8, 12)		2 (2, 3)		12 (10, 15)	
Exercise								
Walking habit	No	30	12 (9, 13)	0.900	3 (2, 3)	0.207	14 (11, 16)	0.548
	Yes	12	11 (9.5, 14)		3 (2.5, 4)		15 (12, 18)	
Fast walking habit	No	22	12 (10, 13)	0.909	3 (2, 3)	0.948	15 (13, 16)	0.980
	Yes	20	11.5 (8, 15)		3 (2, 4)		14.5 (10, 19)	
Eating								
Fast eating speed	No	26	12 (9, 13)	0.825	3 (2, 4)	0.871	15 (11, 16)	0.825
	Yes	16	12 (8, 15)		3 (2, 4)		14.5 (10, 19)	
Short time between dinner and bedtimes (<2 h)	No	10	14.5 (11, 16)	0.016*	4 (3, 4)	<0.01**	18.5 (15, 19)	<0.01**
	Yes	32	11 (8, 13)		2.5 (2, 3)		14 (10, 16)	
Snack after dinner	No	23	12 (9, 13)	1.000	3 (2, 4)	0.732	15 (11, 17)	0.919
	Yes	19	11 (9, 14)		3 (2, 4)		14 (11, 18)	
Breakfast skipping	No	28	12 (9, 14.5)	0.436	3 (2, 4)	0.249	15 (11, 18.5)	0.290
	Yes	14	11 (9, 13)		2.5 (2, 3)		14 (11, 15)	

Values are shown as median, 25th and 75th percentile. *p < 0.05; **p < 0.01
- At 5-months post-entry

At this time point, stress condition scores showed significant correlations or tendencies toward correlation with physical health or urinary examination parameters or nominal variables of the lifestyle questionnaire (Tables 2 and 3, lower sections), as described below. The psychological stress condition score was significantly negatively correlated with systolic blood pressure. This score was also significantly lower in subjects who had reported 1-year body weight change > ± 3 kg or ± 3 kg and reported short times between dinner and bedtimes (≤2 h). The psychological stress condition score also tended to be negatively correlated with urinary sodium and phosphorus, though these relationships were not statistically significant (*p* < 0.10).

The somatic symptom condition score was significantly lower in subjects who had reported 1-year body weight change > ± 3 kg or ± 3 kg. This score was also significantly lower in subjects who had reported short times between dinner and bedtimes (≤2 h). The score tended to be negatively correlated with urinary sodium, though this relationship was not significant (*p* < 0.10).

The combined BJSQ score (psychological stress + somatic symptom condition scores) was significantly negatively correlated with systolic blood pressure and urinary sodium. This score was significantly lower in subjects who had reported 1-year body weight change > ± 3 kg or ± 3 kg, and reported short times between dinner and bedtimes (≤2 h). The combined BJSQ score was significantly negatively correlated with urinary sodium, and tended to be negatively correlated with urinary phosphorus, though this relationship was not statistically significant (*p* < 0.10).

### Stress condition scores at 5 months post-entry vs. selected potential predictors in multiple regression analyses

Parameters showing at least weak correlations (*p* < 0.10) in univariate analysis were targeted for multiple regression analysis (Tables 2 and 3) after confirmation they did not show multicollinearity. In these analyses, stress condition scores at 5 months post-entry were treated as objective variables, and physical health and urinary examination parameters and lifestyle questionnaire items at entry were treated as explanatory variables. Multicollinearity was found for the following parameters: urinary phosphorus was significantly related to urinary sodium (*r* = 0.402, *p* < 0.01), and systolic blood pressure significantly differed between subjects who had reported short times between dinner and bedtimes (≤2 h) and those who did not (*p* = 0.048). After these exclusions for multicollinearity, urinary sodium, 1-year body weight change (> ± 3 kg or ± 3 kg), and times between dinner and bedtimes (short: ≤ 2 h) were found to be significant explanatory variables for psychological stress and somatic symptom condition scores, and for combined BJSQ score at 5 months post-entry (Table 4).

**Table 4:** Standard partial regression coefficients from multiple regression analysis of BJSQ scores at 5 months post-entry

Explanatory variables	B	[95% CI]	Standard error	β	*p* value
A. Psychological stress condition score					
Urinary sodium	−0.57	[−1.09, −0.04]	0.26	−0.29	0.035*
One-year body weight change ≥ 3 kg (Yes = 1)	−2.11	[−3.74, −0.48]	0.81	−0.34	0.013*
Short time between dinner and bedtimes (<2 h) (Yes = 1)	−2.38	[−4.30, −0.45]	0.95	−0.33	0.017*
(adjusted R^2^ = 0.31)					
B. Somatic symptom condition score					
Urinary sodium	−0.18	[−0.34,−0.01]	0.08	−0.28	0.036*
One-year body weight change ≥ 3 kg (Yes = 1)	−0.60	[−1.11,−0.09]	0.25	−0.30	0.023*
Short time between dinner and bedtimes (<2 h) (Yes = 1)	−0.92	[−1.52,−0.31]	0.30	−0.39	<0.01**
(adjusted R^2^ = 0.34)					
C. Combined BJSQ score					
Urinary sodium	−0.74	[−1.36, −0.12]	0.31	−0.30	0.021*
One-year body weight change ≥ 3 kg (Yes = 1)	−2.71	[−4.65, −0.77]	0.96	−0.35	<0.01**
Short time between dinner and bedtimes (<2 h) (Yes = 1)	−3.30	[−5.58, −1.01]	1.13	−0.37	<0.01**
(adjusted R^2^ = 0.37)					

B, non-standardized regression coefficient; β, standardized regression coefficient

R^2^, squared multiple correlation coefficient for the degrees of freedom

CI, confidence interval. *p < 0.05, **p < 0.01

## Discussion

In this study, a number of factors were assessed for their potential to predict susceptibility to occupational stress in female novice nurses. The results suggested that urinary sodium level, 1-year body weight change, and times between dinner and bedtimes (determined at job entry) may have predicted occupational stress at 5 months post-entry.

The psychological stress and somatic symptom condition scores were significantly decreased from baseline at 5 months post-entry (Fig. 2; decreased score represents a higher stress level). The post-entry stress condition scores might have been further decreased if BJSQ data from three subjects who resigned before the end of the analysis period had been included in the analyses. During the first 5 months of their employment at Kagoshima University Hospital, the nurses in this study were primarily involved in training on patient care procedures; therefore, job conditions and content did not differ greatly among study subjects. The nurses were considered an appropriate population in which to assess susceptibility to occupational stress because of the relatively uniform working conditions during this 5-month period. Although the decrease in stress condition scores was speculated to have been primarily due to occupational stress, non-occupational factors may in fact have been involved. Accordingly, two such possible factors were investigated: change in residential location (i.e., moving to a new at the same time as starting a new job) and level of education. The stress condition scores at 5 months post-entry, however, did not differ between subjects who had lived in the same region as the hospital (Kagoshima Prefecture) before job entry and those who moved from elsewhere for the position. This suggested that change of residential location did not affect the mental health condition of the study subjects. Level of education has been reported as a predictor of resignation for novice nurses in Japan [6]. However, in the present study, stress condition scores at 5 months post-entry did not significantly differ between university graduates and vocational college graduates, though university graduates tended to have higher scores (Table 3). From a different standpoint, the present study may not have been sufficiently powered to detect differences between university graduates and technical college graduates.

Urinalysis parameters and nominal variables of the lifestyle questionnaire were also evaluated as stress predictors. The relevant data collections had been considered unproblematic because the procedures were not invasive, yet 16 subjects did not provide a urine sample, and were accordingly excluded from the study (Fig. 1). This may have been explained by the reluctance of some of the nurses to bring urine samples to their workplace in public view, and by possible menstruation-related issues. Urinalysis parameters were nevertheless evaluated in this study despite the lower number of urine samples than expected. Urinary sodium concentration, 1-year (pre-employment) body weight change (≤ ±3 kg), and times between dinner and bedtimes (≤2 h) were targeted for multivariate regression analysis after checking for multicollinearity because they were correlated with the stress condition scores at 5 months post-entry in univariate analyses. Discussion of the results of these multivariate analyses (Table 4) focuses on psychological stress and somatic symptom condition scores, which were closely reflected by the combined BJSQ scores. The univariate analysis results for these parameters were broadly confirmed; urinary sodium concentration, 1-year body weight change (≤ ±3 kg), and times between dinner and bedtimes (≤2 h) were shown to be independent explanatory factors (Table 4). Similar results were obtained for psychological stress and somatic symptom condition scores as objective variables. Urinary sodium was reported to be related to salt intake [18], and high salt intake is reportedly involved in brain renin-angiotensin system activation, not the peripheral system, with resultant increased anxiety, in an animal model [19, 20]. The involvement of sodium intake in susceptibility to occupational stress is a promising area of future research aimed at ameliorating stressful conditions for new employees.

The 1-year body weight change was determined as the self-reported difference from the measurement in the nurses’ previous physical health examination, which they had undergone in their final year of higher education. Decreased energy expenditure and resultant body weight gain could reasonably be expected in that final year, in which they face national nursing examinations and commonly withdraw from sporting or athletic leisure activities. Owing to the varied factors involved, a full discussion of 1-year body weight change is outside the scope of the present study. However, such overeating and/or low energy expenditure-related body weight gains in the final year of high education could induce leptin resistance, which has been linked to adverse mental health outcomes in epidemiological and animal studies [21, 22]. The involvement of body weight change, especially increased body weight, in susceptibility to occupational stress requires further investigation. Nakajima et al. reported that short times between dinner and bedtimes were associated with hyperglycaemia in a general Japanese population, although breakfast skipping was not [23]. Hyperglycaemia is related to insulin resistance, which may relate to mental health conditions underlying susceptibility to occupational stress [24, 25]. Thus, the relationship between eating habit and susceptibility to occupational stress is also a promising area for further investigation in future studies.

Generally, physical health and urinary examination parameters and nominal variables of lifestyle questionnaire were not found to be related to baseline stress conditions scores at job entry, which were relatively high (Tables 2 and 3). However, baseline psychological stress condition and combined BJSQ scores at job entry were significantly low in subjects with a fast eating speed. No such relationship was found for stress condition scores at 5 months post-entry, suggesting that fast eating speed is not a predictive factor for susceptibility to occupational stress.

The present study had three main limitations. First, it was longitudinal, but observational; therefore, caution is required in interpreting cause-and-effect relationships. In addition, the influence of the exclusion (total 20 subjects, see the Fig. 1) during the study on the results is not clear due to the observational study. Moreover, the remained influence of multicollinearity among variables may affect the nature of such findings obtained in the observational study. To address these, investigation in future intervention studies is needed. Second, the analysis population was too small to permit definitive conclusions. This was only a pilot study, and a major study with a larger population is needed to substantiate the results and provide a basis for comprehensive interpretation. However, the statistically significant results obtained in the present study show meaningful findings even from a relatively low number of subjects. Finally, the study population consisted entirely of female novice nurses. Extrapolation to other populations should be considered.

## Conclusions

Urinary sodium, and self-reported (questionnaire response) 1-year body weight change, and times between dinner and bedtimes, are suggested as promising potential predictors of susceptibility to occupational stress. These factors were associated with stress scores at 5 months post-entry, when occupational stress was considered present, and not at baseline. Based on the results, after confirmation of the intervention studies, the subjects with high susceptibility to occupational stress should be managed through the lifestyle corrections.

## Abbreviations

ALT: Alanine transaminase
AST: Aspartate transaminase
BJSQ: Brief job stress questionnaire
Cr: Creatinine
HDL: High-density lipoprotein
LDL: Low-density lipoprotein
SD: Standard deviation
γ-GTP: γ-Glutamyltranspeptidase
